# Negative effects of phosphorus addition outweigh effects of arbuscular mycorrhizal fungi and nitrogen addition on grassland temporal stability in the eastern Eurasian desert steppe

**DOI:** 10.1002/ece3.10368

**Published:** 2023-08-04

**Authors:** Xin Yang, Yuyue Li, Ruize Liang, Bo Ji, Zhanjun Wang, Hongmei Wang, Yue Shen

**Affiliations:** ^1^ College of Forestry and Prataculture Ningxia University Yinchuan China; ^2^ Ningxia Grassland and Animal Husbandry Engineering Technology Research Center Yinchuan China; ^3^ Institute of Forestry and Grassland Ecology Ningxia Academy of Agriculture and Forestry Sciences Yinchuan China

**Keywords:** biomass, diversity, functional group asynchrony, nutrient enrichment, variability

## Abstract

The temporal stability of grassland plant communities is substantially affected by soil nutrient enrichment. However, the potential main and interactive effects of arbuscular mycorrhizal fungi (AMF) and soil nitrogen (N) and phosphorus (P) enrichment on the stability of plant productivity have not yet been clarified. We combined a three‐year in situ field experiment to assess the impacts of soil fertilization and AMF on the stability of plant productivity. P addition decreased the stability of plant productivity by increasing the standard deviation relative to the mean of plant productivity. However, compared to species richness, the stability of C_3_ grasses and other functional groups asynchrony were the most important drivers changing the stability of plant productivity. The negative impacts of P addition overrode the impacts of AMF on the stability of plant productivity. Overall, our study suggests the importance of soil nutrient availability over AMF in terms of shaping the stability of plant productivity. Our results also suggest that three‐year anthropogenic soil nutrient enrichment could reduce the stability of plant communities in grassland regardless of AMF in the P‐limited grassland ecosystem.

## Graphical abstract

P addition decreased the temporal stability of plant productivity by decreasing the standard deviation of plant productivity. However, compared to species richness, the temporal stability of C3 grasses and functional group asynchrony were the most important drivers changing the temporal stability of plant productivity.

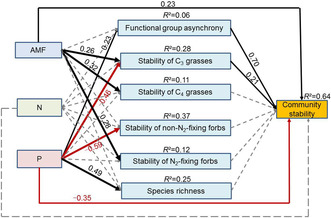

## 1 INTRODUCTION

Determining changes in the temporal stability (here defined as the mean/standard deviation of plant productivity over time; Tilman et al., 2006) of plant communities and the factors that regulate these changes is an essential aspect of ecological research (Chen et al., 2022; Hautier et al., 2020). Terrestrial ecosystems are experiencing the effects of climate change (Lehmann & Rillig, 2014; Rillig Matthias et al., 2019; Song, Wan, et al., 2019; Song, Zong, et al., 2019) and intensive land use (Maestre et al., 2016, 2022; Zhou, Luo, et al., 2019), which can change the stability of plant communities (Chen et al., 2022; Hautier et al., 2020; Ives & Carpenter, 2007; Ma et al., 2017) and thus affect our ability to sustain current ecosystem functioning (Su et al., 2022; Tilman et al., 2014; Valencia et al., 2020).

Plant productivity in terrestrial ecosystems is usually limited by the availability of soil resources (Borer & Stevens, 2022; Eskelinen & Harrison, 2015; Xia & Wan, 2008), and chronic nutrient supply can benefit the production of plant biomass (Borer & Stevens, 2022; Xia & Wan, 2008). Soil nitrogen (N) and phosphorus (P) enrichments shape the stability of aboveground net primary productivity (ANPP) in grassland ecosystems (Carroll et al., 2022; Yang et al., 2022). The global nutrient network experiment (NutNet), conducted in 34 grasslands over 7 years, revealed that N input reduces the stability of ANPP by greatly enhancing inter‐annual variability of annual biomass production (Carroll et al., 2022), and such N‐induced negative effects have also been reported in other studies (Chen et al., 2021; Song, Wan, et al., 2019; Song, Zong, et al., 2019; Yang et al., 2012). The effects of P addition on the stability of ANPP in grassland ecosystems can vary from negative (Carroll et al., 2022; Chen et al., 2021) to positive (Yang et al., 2014) by causing both greater variability and decreased variability in plant productivity (Carroll et al., 2022; Yang, Liu, et al., 2021; Yang, Mariotte, et al., 2021; Yang, Zhang, et al., 2021). Although previous field studies have found that the input of single nutrients (N or P) can strongly influence the stability of ANPP (Carroll et al., 2022; Chen et al., 2021), the interactive effects of N and P inputs on temporal stability in grassland ecosystems require further testing (Carroll et al., 2022; Chen et al., 2021).

Certain soil microorganisms, especially specific soil mutualists, are known to be important in temporally stabilizing plant communities, as shown in several field and greenhouse experiments (Wagg et al., 2021; Yang, 2021; Yang, Liu, et al., 2021; Yang, Mariotte, et al., 2021; Yang, Shen, et al., 2018; Yang, Wagg, et al., 2018; Yang, Zhang, et al., 2021). Arbuscular mycorrhizal fungi (AMF) are a type of fungus that forms a symbiotic relationship with host plants, and enhance the stability of plant productivity (Jia et al., 2022; Kang et al., 2020; Yang et al., 2014; Yang, Liu, et al., 2021; Yang, Mariotte, et al., 2021; Yang, Zhang, et al., 2021). Jia et al. (2021) reported that AMF can increase plant diversity (according to the Shannon–Wiener diversity index) and improve the stability of ANPP in an arid grassland, while the input of N had opposite effects in this environment. Additionally, soil availability of N and P is known to alter the structure and function of AMF in grassland ecosystems (Han et al., 2020; Jiang et al., 2018; Treseder, 2004), potentially influencing the stabilizing effect of fungi on plant communities (Chen et al., 2021). However, field evidence of the independent or interactive effects of AMF and soil enrichment with N and P on the stability of plant communities remains limited.

Previous in situ studies revealed that the stability of ANPP in grasslands could be modified by the richness of plant species (Zhang et al., 2018), the asynchrony of plant species and/or functional groups (Huang et al., 2020; Sasaki et al., 2019; Xu et al., 2015) and the stability of plant populations and/or functional groups (Jiang et al., 2021). Five‐year‐long study indicated that N input alone had a neutral impact on the stability of ANPP in meadow grassland (Kang et al., 2020). Plant species richness can weaken the effect of inter‐annual variability on a plant community in terms of resistance/resilience enhancement that can come with greater species richness instead (Hautier et al., 2015). High plant species richness can enhance plant community resistance/resilience, which can then decrease inter‐annual variability in productivity (Yang, Liu, et al., 2021; Yang, Mariotte, et al., 2021; Yang, Zhang, et al., 2021). Thus, higher plant species richness in grassland ecosystems can increase the stability of ANPP due to asynchronous responses of plant species and/or functional groups when environmental fluctuations occur (Huang et al., 2020). The asynchrony of plant functional groups, which is defined as different functional groups responding dissimilarly through time (Huang et al., 2020; Ma et al., 2022; Wilcox et al., 2017), is found as a vital driver of stability of plant productivity (Leps et al., 2019; Ma et al., 2022). Wilcox et al. (2017) found that plant asynchrony increased the stability of plant productivity across five continents.

The stability of specific plant population or functional groups is vital for stabilizing plant communities in grassland ecosystems (Jiang et al., 2021; Šmilauer et al., 2021). In a previous in situ experiment, Yang, Liu, et al. (2021), Yang, Mariotte, et al. (2021) and Yang, Zhang, et al. (2021) showed that soil N enrichment and AMF independently alter the stability of ANPP by modifying the stability of dominant species but not plant species richness. Although the factors that determine the stability of plant communities are well‐documented (Gross et al., 2014; Ives & Carpenter, 2007; Pimm, 1984), the relative importance of these driving factors in maintaining the stability of ANPP in response to soil nutrient enrichment and AMF remains poorly understood (Yang, Shen, et al., 2018; Yang, Wagg, et al., 2018).

The desert steppe within the eastern Eurasian steppe, which comprises more than 25% of the grassland area in China, is an exceedingly xeric grassland type (Kang et al., 2007). Previous studies found that plant production is extremely limited by N and P in this grassland ecosystem (Ma et al., 2020; Yang, Liu, et al., 2021; Yang, Mariotte, et al., 2021; Yang, Zhang, et al., 2021). Extreme soil nutrient limitation may hamper the mutualistic relationship between host plant and AMF, and then impairs the benefits of AMF for their host plants (Albornoz et al., 2021; Lambers et al., 2022). In the present study, we performed an in situ experiment to determine how AMF affects the stability of ANPP under N, P and N + P (combined) treatments in a desert steppe. We tested three hypotheses: (1) soil nutrient availability alone has a larger effect on plant community stability than AMF alone; (2) soil nutrient availability changes the stability of plant community through altering variability of plant productivity; (3) asynchrony across plant functional groups, as well as stability of dominant functional groups (e.g. C_3_ grasses) have the greatest influence on overall plant community stability.

## 2 MATERIALS AND METHODS

### 2.1 Study site

Our in situ experiment site was located in Ningxia, Northwest China (37.7562° N, 107.2765° E; 1348 m asl). This region has a temperate continental climate with a mean annual temperature (MAT) of 8.1°C and a long‐term mean annual precipitation (MAP) of 282.5 mm (from 1980 to 2020), of which >87.29% (~246.6 mm) occurs from 1 April to 30 September (Appendix S1). The type of soil in the region is light sierozem with a pH of 8.76, soil organic carbon concentration of 2.02 mg kg^−1^ and soil inorganic N and available plant P (Olsson‐P) concentrations of 6.68 and 2.39 mg kg^−1^, respectively. The vegetation type is classified as temperate desert steppe, and the dominant plant functional group is C_3_ grasses (mainly including *Stipa breviflora* and *Agropyron mongolicum*). Common plant species include *Cleistogenes squarrosa* (a perennial C_4_ grass), *Astragalus melilotoides* (a perennial legume), *Polygala tenuifolia, Euphorbia esula*, *Torularia humilis* and *Heteropappus altaicus* (four types of perennial forbs) (Appendix S2). Except for Chenopodiaceae species, all plant species in our study site are colonized by AMF (Yang, Liu, et al., 2021; Yang, Mariotte, et al., 2021; Yang, Zhang, et al., 2021). This region has been openly grazed since 2002 until it was fenced off to exclude livestock in 2017.

### 2.2 Experimental design

The in situ experimental design was previously reported by Yang, Liu, et al. (2021), Yang, Mariotte, et al. (2021) and Yang, Zhang, et al. (2021). Briefly, this study had a random block experimental design with five blocks and eight treatments, including a control (without N and P input), N input (N treatment; 10 g of N m^−2^ year^−1^ applied as ammonium nitrate), P input (P treatment; 10 g of P m^−2^ year^−1^ applied as superphosphate) and combined N and P input (N + P treatment; 10 g of N+ 10 g of P m^−2^ year^−1^) in no fungicide addition and fungicide addition plots. Therefore, eight experimental treatments were randomly assigned to eight experimental plots in each block. Each experimental plot (2.2 m × 2.2 m) was surrounded by 1.0 m buffer walkways. The amounts of N and P applied exceeded the actual levels of atmospheric nutrient deposition in China but corresponded to the actual deposition of N and P in the agro‐pasture ecotone in this region (Ma et al., 2020). Fungicide addition treatment was applied in each growing season using benomyl (6 g of effective constituent 10 L^−1^ of water m^−2^ applied per 2 weeks), while no fungicide addition treatment involved the application of equal amounts of water in each two‐week period. Benomyl was used as a fungicide because it effectively reduces mycorrhizal root colonization of plants as well as extraradical hyphal density, but it has negligible effects on host plants and non‐target soil bacteria, fungi and protozoa in grassland ecosystems (Jia et al., 2022; Yang, Liu, et al., 2021; Yang, Mariotte, et al., 2021).

### 2.3 Plant, soil and AM fungal traits

Two 0.5 m × 0.5 m quadrats were used randomly within each plot in mid‐August (peak biomass period) in 2019–2021. The shoot biomass of each plant species in each plot was clipped to the soil surface, classified into plant species and then oven‐dried at 65°C for nearly 72 h and weighed. We combined all shoot biomass of two quadrants in each experimental plot and defined ANPP (g m^−2^) as the total dry weights of each plant species. All plant species were also classified into plant functional groups (C_3_ grasses, C_4_ grasses, non‐N_2_‐fixing forbs and N_2_‐fixing forbs; Appendix S2). ANPP data for plant species and functional groups in 2019 were previously reported (Yang, Liu, et al., 2021; Yang, Mariotte, et al., 2021; Yang, Zhang, et al., 2021), whereas data for 2020 and 2021 are reported for the first time.

We randomly obtained three 10 cm deep soil cores in each plot in mid‐August 2019–2021. We mixed soil samples and then sieved through a 2 mm mesh to separate the soil and the mixed roots, after which the soil samples were immediately frozen for neutral lipid fatty acids (NLFA) analysis (Andrino et al., 2021; Ngosong et al., 2012; Olsson & Lekberg, 2022). The inorganic N content of soil was analysed using a flow injection auto analyzer and the available P content of soil was analysed using the Olsen method (Chen et al., 2010). The mixed root samples in each plot were cut to a length of 0.5 cm and mixed, after which they were cleaned with 10% potassium hydroxide (KOH) at 90°C for 2 h, acidified in 2% hydrochloric acid (HCL) for 5 min and then stained with 0.05% trypan blue (Kormanik & Mcgraw, 1982). The colonization of mycorrhizal roots was then evaluated with a grid intersection method using a microscope (Giovannetti & Mosse, 1980).

### 2.4 Assessment of temporal stability

Stability was calculated at the plant functional group and community level shoot biomass as the ratio of *μ*/*σ*, where *μ* is the mean shoot biomass of each functional group or total ANPP in each experimental plot and σ is the standard deviation (SD) of *μ* from 2019 to 2021 (Ma et al., 2017; Šmilauer et al., 2021). The asynchrony of the functional groups (1 − *φ*) was calculated as follows:

$$1-\varphi =1-\frac{\sigma^{2}}{{\left(\sum_{i=1}^{s}\sigma_{i}\right)}^{2}}$$

where *φ* is plant functional group synchrony, *σ* ^2^ is the variance of the ANPP and *σ*_*i*_ is the SD of the shoot biomass of the *i*th PFG in an experimental plot.

### 2.5 Data analyses

To assess the impacts of N and P inputs and fungicide application on temporal stability at functional group and community levels and functional group asynchrony, three‐way ANOVA with three fixed factors (N and P inputs and fungicide application) and one random factor (blocks) were conducted. Repeated measures ANOVA were conducted to evaluate the effects of N input, P inputs, fungicide application and their interaction on plant shoot biomass and diversity, soil available nutrients and AMF traits. The normality and homogeneity of variances were verified for all data using Kolmogorov–Smirnov and Levene tests, respectively. Three‐way ANOVA and repeated measures ANOVA were performed using SPSS (version 22.0; IBM). Structural equation modelling (SEM) was performed to determine how mycorrhizal suppression and N and P inputs altered the stability of the plant community. We use a *χ* ^2^ test, the Akaike information criterion (AIC) and the root mean square error (RMSE) of the approximation to test the fitness of SEM model. SEM was conducted using Amos (version 21; IBM).

## 3 RESULTS

### 3.1 Effects of fungicide, N and P on plant community stability

**FIGURE 1:**
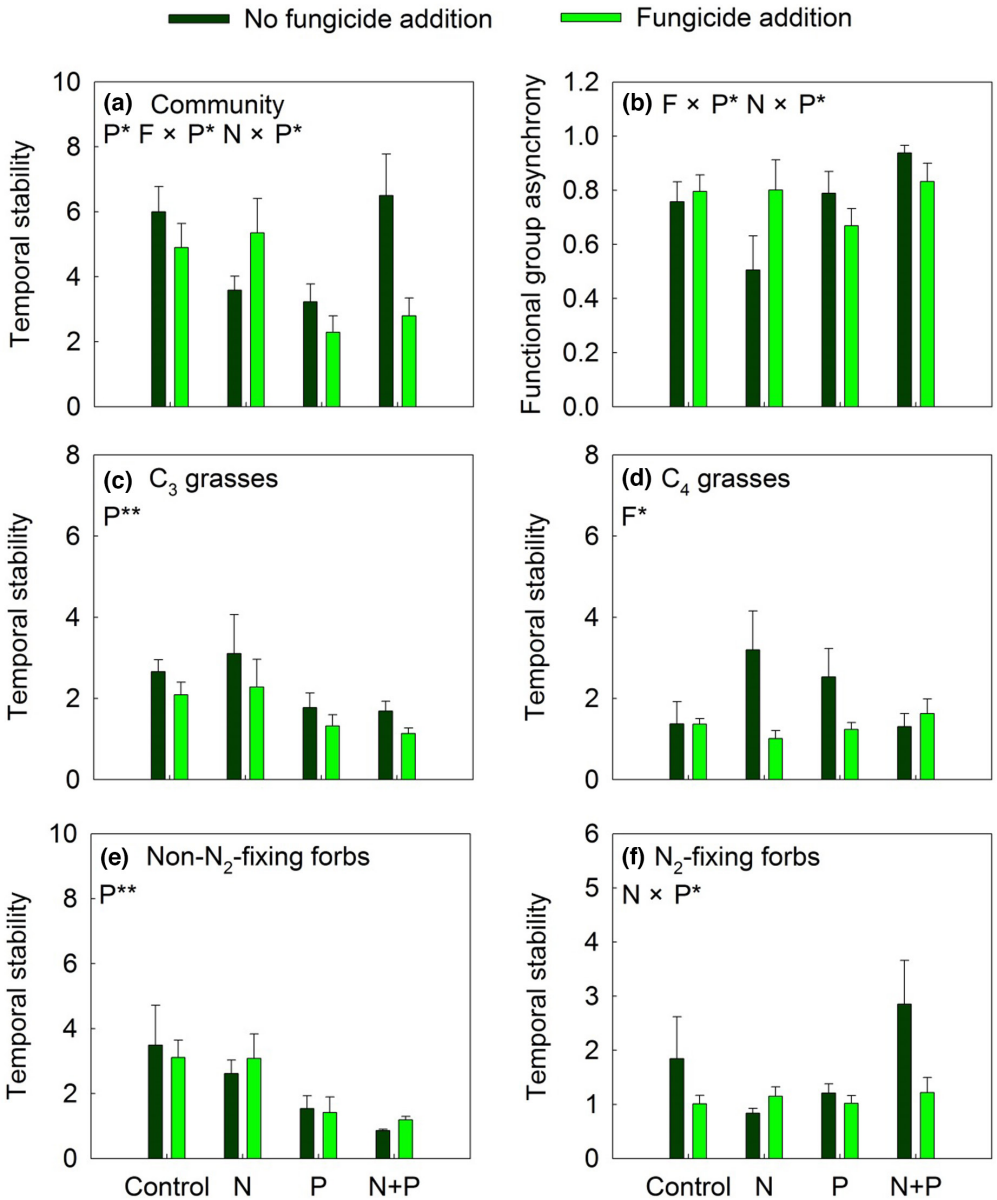
Response of temporal stability of plant community (a) and functional group asynchrony (b), and C_3_ grasses (c), C_4_ grasses (d), Non‐N_2_‐fixing forbs (e) and N_2_‐fixing forbs (f) to nitrogen addition (N) and phosphorus addition (P) in no fungicide addition (dark green bars) and fungicide addition (light green bars) plots from 2019 to 2021. F, fungicide application. Data are presented as means + SE. *p < .05; **p < .01; NS *p* > .05.

**FIGURE 2:**
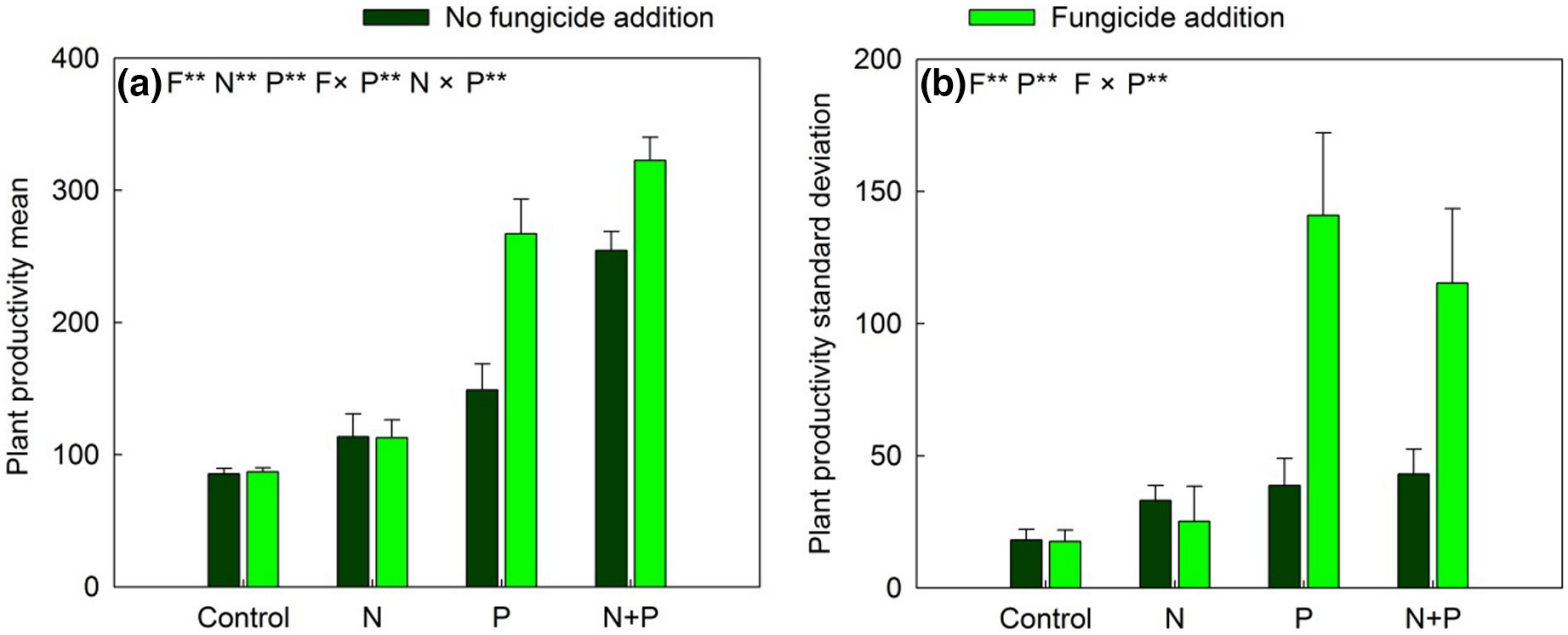
Response of the temporal mean (a) and standard deviation (b) of plant productivity to nitrogen addition (N) and phosphorus addition (P) in no fungicide addition (dark green bars) and fungicide addition (light green bars) plots from 2019 to 2020. F, fungicide application. Data are presented as means + SE. *p < .05; **p < .01; NS *p* > .05.

Fungicide and P inputs interactively affected the stability of ANPP (F × P: *F*_1,28_ = 5.70, *p* < .05; Table 1 and Figure 1a). Fungicide addition did not change the stability of ANPP in control plots; however, compared with P+ no‐fungicide treatment, P+ fungicide significantly decreased the stability of ANPP by 91.53% under P input conditions (Figure 1a). N and P inputs antagonistically affected the stability of ANPP (N × P: *F*_1,28_ = 6.66, *p* < .05; Table 1 and Figure 1a). P addition significantly reduced the stability of ANPP in control, but it did not have this effect in N addition plots (Figure 1a). At the plant community level, fungicide addition significantly reduced functional groups asynchrony by 16.46% only in +P and +N+P plots (Figure 1b). At the plant functional group level, P input alone reduced the stability of C_3_ grasses and non‐N_2_‐fixing forbs, but had no significant effect on that of C_4_ grasses (Figure 1c–e). However, fungicide addition effectively reduced the stability of C_4_ grasses across all fertilization treatments (Figure 1d). N and P inputs interactively altered the stability of N_2_‐fixing forbs (Figure 1f); P addition suppressed and enhanced the stability of N_2_‐fixing forbs in the control and +N plots, respectively (Figure 1f). P and N inputs alone significantly enhanced the temporal mean of ANPP (by 148.76% and 36.45%, respectively), whereas fungicide addition increased the temporal mean ANPP in +P and +N+P plots (Figure 2a). Fungicide addition also substantially increased the standard deviation of ANPP in P‐addition plots (Figure 2b).

**TABLE 1:** Analysis of variance for the effects of fungicide application (F), nitrogen addition (N) and phosphorus addition (P) on the stability of plant functional group and plant community.

Effects	df	Stability of community biomass		Stability of C_3_ grasses		Stability of C_4_ grasses		Stability of non‐N_2_‐fixing forbs		Stability of N_2_‐fixing forbs		Functional groups asynchrony	
		F	P	F	P	F	P	F	P	F	P	F	P
Block	4, 28	0.96	0.44	0.21	0.93	1.17	0.34	0.14	0.97	1.00	0.42	0.78	0.54
F	1, 28	3.20	0.08	2.81	0.10	**5.03**	**0.03**	0.03	0.87	3.77	0.06	1.15	0.29
N	1, 28	0.66	0.42	0.07	0.80	0.20	0.66	0.99	0.33	0.65	0.43	1.64	0.21
P	1, 28	**5.09**	**0.03**	**8.70**	**0.01**	0.03	0.86	**16.11**	**<0.01**	1.47	0.24	1.11	0.30
F × N	1, 28	0.00	0.97	0.06	0.80	0.16	0.69	0.51	0.48	0.06	0.81	0.26	0.62
F × P	1, 28	**5.70**	**0.02**	0.07	0.79	0.76	0.39	0.00	0.95	1.17	0.29	**6.09**	**0.02**
N × P	1, 28	**6.66**	**0.02**	0.41	0.53	2.67	0.11	0.00	1.00	**5.05**	**0.03**	**4.51**	**0.04**
F × N × P	1, 28	4.41	0.12	0.01	0.91	2.22	0.18	0.05	0.83	**4.61**	**0.04**	0.48	0.49

*Note*: ANPP and the aboveground biomass of plant functional group all have the same df. Bold values are significant at *p* < .05.

**FIGURE 3:**
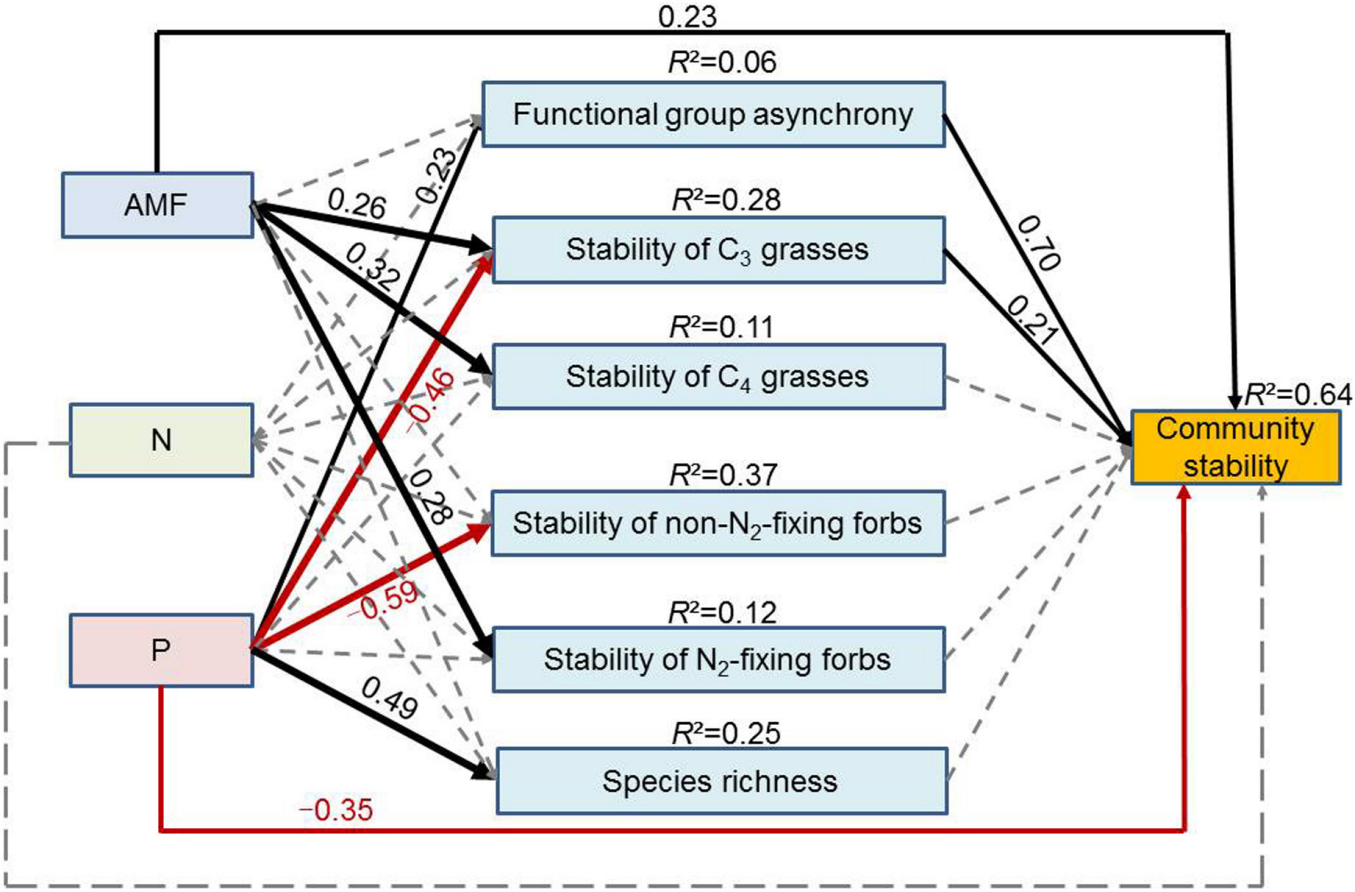
The results of structural equation modelling (SEM) show the relationships among nitrogen addition (N), phosphorous addition (P), arbuscular mycorrhizal fungi (AMF), and stability of plant functional groups and plant community. Numbers adjacent to arrows are standardized path coefficients and suggest the effect size of the relationship. The proportion of variance explained (*R* ^2^) appears alongside the response variables in the model. Arrows indicate significant (black and solid; red and solid) and non‐significant (grey and dashed) relationships. Goodness‐of‐fit statistics for each model are shown below the model. *χ* ^2^ = 27.094, *p* = .077, df = 18; RMSEA = 0.114, AIC = 101.094.

According to the best‐fitting SEM, AMF and P input directly affected the stability of ANPP and the negative effect of P input was stronger than the positive effect of AMF. Asynchrony of functional groups and the stability of C_3_ grasses (i.e. the dominant functional group) were the best predictors of plant community stability, which was itself indirectly affected by the addition of P (Figure 3). The stability of N_2_‐fixing forbs, non‐N_2_‐fixing forbs and C_4_ grasses did not affect the stability of ANPP (Figure 3). P addition also increased species richness, but this did not change the stability of ANPP (Figure 3).

### 3.2 Effects of fungicide, N and P on plant, soil and AMF properties

**FIGURE 4:**
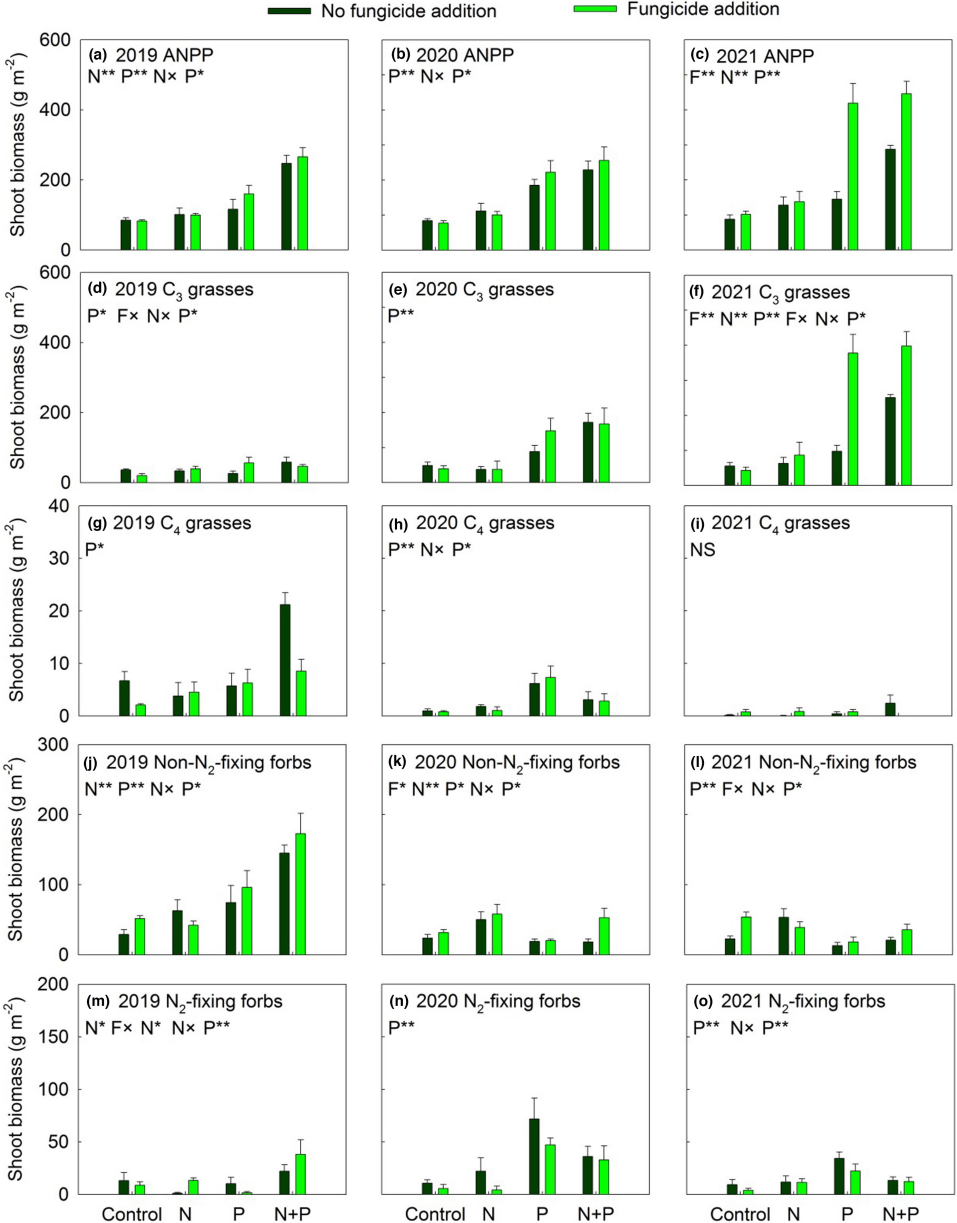
Response of above‐ground net primary productivity (ANPP, a, b and c) and plant functional groups (d–o) to nitrogen addition (N) and phosphorus addition (P) in no fungicide addition (dark green bars) and fungicide addition (light green bars) plots from 2019 to 2020. F, fungicide application. Data are presented as means + SE. *p < .05; **p < .01; NS *p* > .05.

**FIGURE 5:**
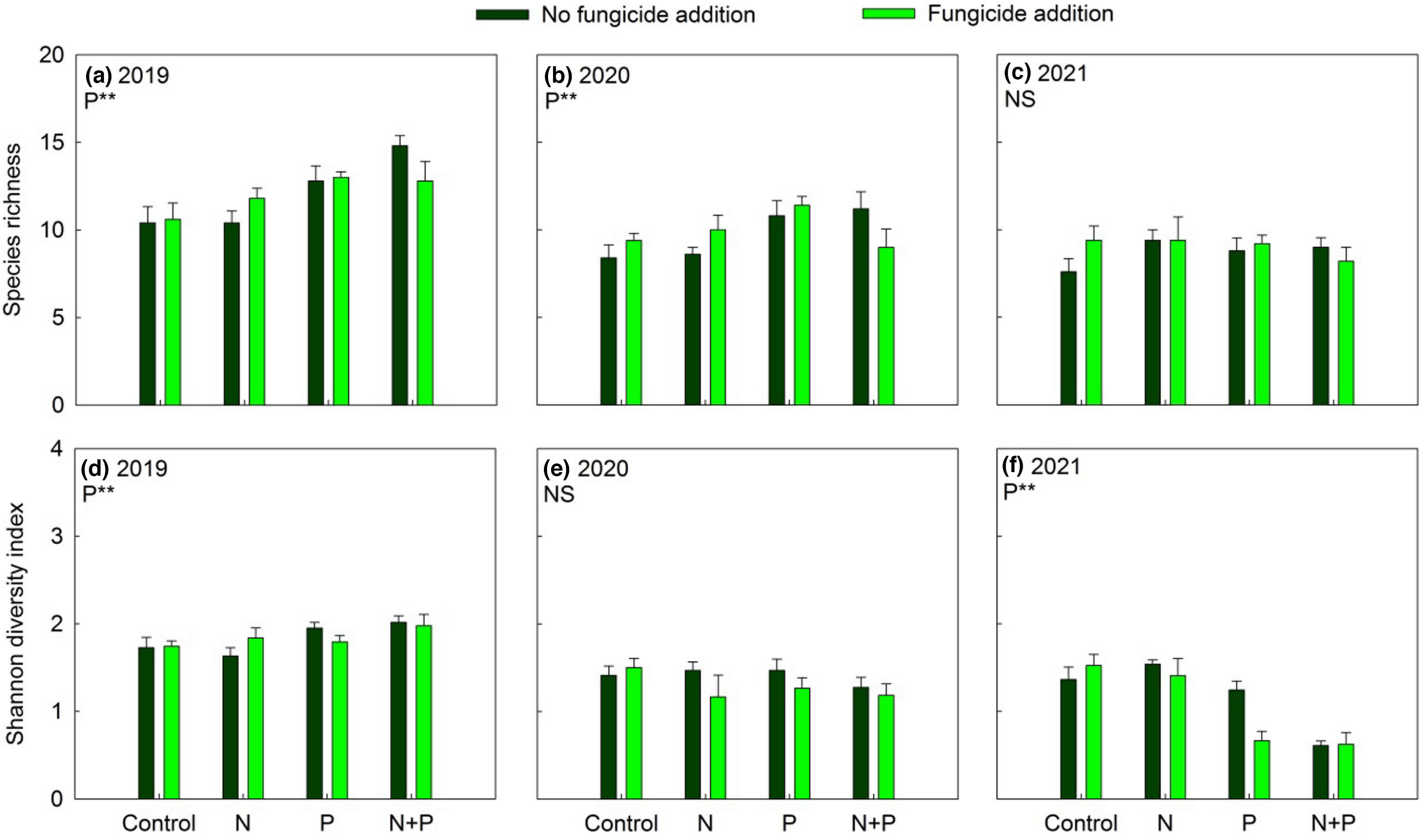
Response of species richness (a–c), Shannon diversity index (d–f) to nitrogen addition (N) and phosphorus addition (P) in no fungicide addition (dark green bars) and fungicide addition (light green bars) plots from 2019 to 2020. Data presented as means + SE. *p < .05; **p < .01; NS *p* > .05.

Throughout the 3 years of the study, both N and P inputs significantly increased the shoot biomass of most plant functional groups and ANPP (all *p* < .05; Appendix S3 and Figure 4). The positive effects of N and P inputs on the shoot biomass of C_4_ grasses and non‐N_2_‐fixing forbs were more evident in 2019 than in 2020 and 2021 (Appendix S3 and Figure 4g–j). The P input significantly increased the shoot biomass of N_2_‐fixing forbs only in 2020 and 2021 (Figure 4n,o). Fungicide addition significantly increased the shoot biomass of C_3_ grasses and ANPP in +P plots, especially in 2021 (Figure 4c,f). In all 3 years, P input alone significantly increased plant species richness, while P input with mycorrhizal suppression did not change plant species richness (Appendix S4 and Figure 5).

N input alone increased soil inorganic N in 2020 and 2021 (Appendix S6a–c), and P addition improved soil available P across all 3 years (Appendix S6d–f). Fungicide application alone did not alter soil available P and soil inorganic N in the present study (all *p* > .05; Appendix S6). In all 3 years, fungicide and P applications effectively reduced mycorrhizal root colonization and AMF NLFA concentration at the plant community level (Appendices S5, S7 and S8). Across all experimental treatments, fungicide addition significantly reduced mycorrhizal root colonization in 2019, 2020 and 2021 by 13.68%, 14.31% and 15.57%, respectively (Appendix S7). Moreover, fungicide addition significantly reduced AMF NLFA concentration in 2019, 2020 and 2021 by 19.56%, 37.76% and 32.70%, respectively (Appendix S8).

## 4 DISCUSSION

### 4.1 Effects of N, P and fungicide inputs on the stability of ANPP

From the field study, the stability of plant community ANPP was substantially decreased by soil enrichment with N and P, which was in accordance with previous studies of different native grassland ecosystems (Yang et al., 2012; Zhang et al., 2016) and a global net experiment (Carroll et al., 2022). P addition affected the stability of ANPP mainly by increasing its standard deviation over its temporal mean in our field study. Compared to the first 2 years (2019 and 2020), P addition greatly increased ANPP by enhancing the shoot biomass of C_3_ grasses in the third year (2021) and then lead to a disproportionate increase in standard deviation of ANPP in our field study. According to previous work, the negative impacts of P addition on plant community stability were also shown by the increase in standard deviation in a global nutrient network experiment over 7 years (Carroll et al., 2022). In the present study, an antagonistic interaction between AMF and P input affected plant community stability. When P was not added, mycorrhizal suppression did not change plant community stability, which was comparable with that in no fungicide addition plots. However, when P was added, it overrode the effects of AMF on plant community stability, presumably because AMF fungi markedly reduced the standard deviation of plant productivity in the P input plots. In accordance with a previous study (Yang et al., 2014), we found that AMF has the potential to enhance the stability of plant productivity under P input conditions.

Carroll et al. (2022) found that N input had a destabilizing impact on plant community stability in grassland ecosystems across the world. However, in our field study, compared with P addition, N supply alone had a negligible effect on plant productivity and stability. Previous work found that sufficient precipitation in the growing season could improve the positive influence of N fertilizer on plant productivity in N‐limited grassland (Lee et al., 2010). However, our study experienced a drought early in the growing season from 2020 to 2021, which could have limited the effects of N input on plant productivity in our study. Water limitation may hamper the effect of N input on plant productivity in our field study.

Plant diversity and/or the abundance of functional groups are known determinants of plant productivity stability (Huang et al., 2020; Ma et al., 2022; Zhou, Li, et al., 2019). We found that the stability of C_3_ grass is a major contributor to plant community stability. Many of the C_3_ grass species in our study site are relatively sensitive to soil P enrichment because the desert steppe is a P‐impoverished ecosystem (Yang, Liu, et al., 2021; Yang, Mariotte, et al., 2021; Yang, Zhang, et al., 2021). Our SEM model also indicated that functional group asynchrony was positively related to the stability of plant productivity. Specifically, P input increased plant functional groups asynchrony when AMF was intact, and the P addition treatments reduced the stability of C_3_ grasses and non‐N_2_‐fixing forbs but stabilized that of C_4_ grasses and N_2_‐fixing forbs. This could explain the high degree of functional groups asynchrony observed in the present study. Notably, we found a synergistic interaction between P input and AMF, where AMF increased functional groups asynchrony under P input conditions. Different plant functional groups have varying mycorrhizal growth responses, in part because of the differential ability to supply carbon to AMF under P addition. Functional groups in plant communities have different mycorrhizal growth responses and the ability of C supply for AMF under P input (Hoeksema et al., 2010; Lin et al., 2015). Specifically, C supply to AMF can be reduced for certain functional groups under P input, causing asynchrony in ANPP across functional groups. Thus, the interaction between soil nutrient availability and AMF may alter the stability of plant productivity, particularly in arid grassland ecosystems.

### 4.2 Effects of N, P and fungicide inputs on environmental variables

Previous studies have revealed that soil nutrient enrichment alters plant species richness, thereby changing the stability of plant productivity (Hautier et al., 2015; Huang et al., 2020; Xu et al., 2021; Yang et al., 2012). Zhou et al. (2020) also showed that a positive relationship exists between plant productivity stability and plant species richness. However, in the present study, we found no positive diversity–stability relationship. This may have been due to the low variation in plant species richness (an increase of only 1–3 plant species) in the P input plots. Moreover, two N_2_ fixing forbs, *Oxytropis racemosa* and *Gueldenstaedtia verna*, dominated the P input plots, but accounted for a low proportion of ANPP and had lower stability than other functional groups. Similarly, previous field experiments have found that plant species richness is not the primary driver of stability of ANPP (Ma et al., 2021; Xu et al., 2015).

We showed that P input and fungicide application suppressed mycorrhizal root colonization and its NLFA concentration in 2019–2021, which aligns with previous work in native grassland ecosystems (Qiao et al., 2019; Yang, Shen, et al., 2018; Yang, Wagg, et al., 2018). Furthermore, the application of benomyl did not alter the NLFA concentrations of other microorganism groups (e.g. pathogenic fungi) in the present study, confirming that this fungicide effectively suppresses the structure of AMF without affecting other microorganisms, which is consistent with previous field studies conducted in a temperate steppe (Yang et al., 2014) and desert steppe (Yang, Liu, et al., 2021; Yang, Mariotte, et al., 2021; Yang, Zhang, et al., 2021).

Our in situ study revealed that the negative effects of P addition outweigh the effects of AMF and N input on plant community stability in this P‐impoverished grassland ecosystem. Yang et al. (2014) found that P supplementation tended to increase plant productivity stability when AMF was intact; however, our field study revealed that the addition of P had the opposite effect, and this was supported by the results of global NutNet study (Carroll et al., 2022). The different responses of plant stability to soil N and P enrichment and AMF suggest that these factors may have interacting effects on plant productivity stability. However, our in situ field study lasted only 3 years, a relatively short duration for analyzing plant stability (Xu et al., 2021). Understanding of plant stability to long‐term fertilizer and fungicide input is scarce (Carroll et al., 2022), and requires further study.

## 5 CONCLUSIONS

In conclusion, our study indicated that P inputs decreased plant productivity stability by increasing the standard deviation of plant productivity directly and decreasing the stability of the dominant functional group (C_3_ grasses). Furthermore, we found that AMF have potential effects on plant productivity stability associated with P input in an arid grassland ecosystem. The negative effects of P input overrode the effects of AMF and N input on plant community stability. Soil fertility and AMF are important for the stability of ANPP in grassland ecosystems; therefore, our findings improve our understanding of the response of plant community dynamics to AMF and anthropogenic soil nutrient enrichment.

## AUTHOR CONTRIBUTIONS

**Xin Yang:** Investigation (lead); methodology (lead); writing – original draft (lead). **Yuyue Li:** Formal analysis (supporting); investigation (supporting); methodology (supporting). **Ruize Liang:** Formal analysis (equal); software (equal). **Bo Ji:** Formal analysis (equal); funding acquisition (equal); investigation (equal); visualization (equal); writing – original draft (equal). **Zhanjun Wang:** Data curation (equal); formal analysis (equal); funding acquisition (equal); validation (equal); writing – original draft (equal). **Hongmei Wang:** Data curation (equal); software (equal); supervision (equal); validation (equal). **Shen Yue:** Investigation (lead); project administration (lead); visualization (lead); writing – original draft (lead); writing – review and editing (lead).

## CONFLICT OF INTEREST STATEMENT

The authors declare no conflict of interest.

## Supporting information


Appendix S1–S8

## Data Availability

Data available from Zenodo under https://doi.org/10.5281/zenodo.7020845.

## References

[ece310368-bib-0001] Albornoz, F. E. , Dixon, K. W. , & Lambers, H. (2021). Revisiting mycorrhizal dogmas: Are mycorrhizas really functioning as they are widely believed to do? Soil Ecology Letters, 3(1), 73–82. 10.1007/s42832-020-0070-2

[ece310368-bib-0002] Andrino, A. , Guggenberger, G. , Sauheitl, L. , Burkart, S. , & Boy, J. (2021). Carbon investment into mobilization of mineral and organic phosphorus by arbuscular mycorrhiza. Biology and Fertility of Soils, 57(1), 47–64. 10.1007/s00374-020-01505-5

[ece310368-bib-0003] Borer, E. T. , & Stevens, C. J. (2022). Nitrogen deposition and climate: An integrated synthesis. Trends in Ecology & Evolution, 37(6), 541–552. 10.1016/j.tree.2022.02.013 35428538

[ece310368-bib-0004] Carroll, O. , Batzer, E. , Bharath, S. , Borer, E. T. , Campana, S. , Esch, E. , Hautier, Y. , Ohlert, T. , Seabloom, E. W. , Adler, P. B. , Bakker, J. D. , Biederman, L. , Bugalho, M. N. , Caldeira, M. , Chen, Q. , Davies, K. F. , Fay, P. A. , Knops, J. M. H. , Komatsu, K. , … MacDougall, A. S. (2022). Nutrient identity modifies the destabilising effects of eutrophication in grasslands. Ecology Letters, 25(4), 754–765. 10.1111/ele.13946 34957674

[ece310368-bib-0005] Chen, C. R. , Phillips, I. R. , Wei, L. L. , & Xu, Z. H. (2010). Behaviour and dynamics of di‐ammonium phosphate in bauxite processing residue sand in Western Australia‐II. Phosphorus fractions and availability. Environmental Science and Pollution Research, 17(5), 1110–1118. 10.1007/s11356-009-0268-4 19941089

[ece310368-bib-0006] Chen, Q. , Wang, S. , Seabloom, E. W. , MacDougall, A. S. , Borer, E. T. , Bakker, J. D. , Donohue, I. , Knops, J. M. H. , Morgan, J. W. , Carroll, O. , Crawley, M. , Bugalho, M. N. , Power, S. A. , Eskelinen, A. , Virtanen, R. , Risch, A. C. , Schütz, M. , Stevens, C. , Caldeira, M. C. , … Hautier, Y. (2022). Nutrients and herbivores impact grassland stability across spatial scales through different pathways. Global Change Biology, 28(8), 2678–2688. 10.1111/gcb.16086 35038782

[ece310368-bib-0007] Chen, Z. , Xiong, P. , Zhou, J. , Lai, S. , Jian, C. , Xu, W. , & Xu, B. (2021). Effects of plant diversity on semiarid grassland stability depends on functional group composition and dynamics under N and P addition. Science of the Total Environment, 799, 149482. 10.1016/j.scitotenv.2021.149482 34365257

[ece310368-bib-0008] Eskelinen, A. , & Harrison, S. P. (2015). Resource colimitation governs plant community responses to altered precipitation. Proceedings of the National Academy of Sciences of the United States of America, 112(42), 13009–13014. 10.1073/pnas.1508170112 26438856 PMC4620885

[ece310368-bib-0009] Giovannetti, M. , & Mosse, B. (1980). An evaluation of techniques for measuring vesicular arbuscular mycorrhizal infection in roots. New Phytologist, 84(3), 489–500. 10.1111/j.1469-8137.1980.tb04556.x

[ece310368-bib-0010] Gross, K. , Cardinale, B. J. , Fox, J. W. , Gonzalez, A. , Loreau, M. , Polley, H. W. , Reich, P. B. , & van Ruijven, J. (2014). Species richness and the temporal stability of biomass production: A new analysis of recent biodiversity experiments. American Naturalist, 183(1), 1–12. 10.1086/673915 24334731

[ece310368-bib-0011] Han, Y. , Feng, J. , Han, M. , & Zhu, B. (2020). Responses of arbuscular mycorrhizal fungi to nitrogen addition: A meta‐analysis. Global Change Biology, 26(12), 7229–7241. 10.1111/gcb.15369 32981218

[ece310368-bib-0012] Hautier, Y. , Tilman, D. , Isbell, F. , Seabloom Eric, W. , Borer Elizabeth, T. , & Reich Peter, B. (2015). Anthropogenic environmental changes affect ecosystem stability via biodiversity. Science, 348(6232), 336–340. 10.1126/science.aaa1788 25883357

[ece310368-bib-0013] Hautier, Y. , Zhang, P. , Loreau, M. , Wilcox, K. R. , Seabloom, E. W. , Borer, E. T. , Byrnes, J. E. K. , Koerner, S. E. , Komatsu, K. J. , Lefcheck, J. S. , Hector, A. , Adler, P. B. , Alberti, J. , Arnillas, C. A. , Bakker, J. D. , Brudvig, L. A. , Bugalho, M. N. , Cadotte, M. , Caldeira, M. C. , … Wang, S. (2020). General destabilizing effects of eutrophication on grassland productivity at multiple spatial scales. Nature Communications, 11(1), 5375. 10.1038/s41467-020-19252-4 PMC7585434 33097736

[ece310368-bib-0014] Hoeksema, J. D. , Chaudhary, V. B. , Gehring, C. A. , Johnson, N. C. , Karst, J. , Koide, R. T. , Pringle, A. , Zabinski, C. , Bever, J. D. , Moore, J. C. , Wilson, G. W. , Klironomos, J. N. , & Umbanhowar, J. (2010). A meta‐analysis of context‐dependency in plant response to inoculation with mycorrhizal fungi. Ecology Letters, 13(3), 394–407. 10.1111/j.1461-0248.2009.01430.x 20100237

[ece310368-bib-0015] Huang, M. , Liu, X. , & Zhou, S. (2020). Asynchrony among species and functional groups and temporal stability under perturbations: Patterns and consequences. Journal of Ecology, 108(5), 2038–2046. 10.1111/1365-2745.13418

[ece310368-bib-0016] Ives, A. R. , & Carpenter, S. R. (2007). Stability and diversity of ecosystems. Science, 317(5834), 58–62. 10.1126/science.1133258 17615333

[ece310368-bib-0017] Jia, Y. , Walder, F. , Wagg, C. , & Feng, G. (2021). Mycorrhizal fungi maintain plant community stability by mitigating the negative effects of nitrogen deposition on subordinate species in Central Asia. Journal of Vegetation Science, 32(1), e12944. 10.1111/jvs.12944

[ece310368-bib-0018] Jia, Y. , Zhang, T. , Walder, F. , Sun, Y. , Shi, Z. , Wagg, C. , Tian, C. , & Feng, G. (2022). Can mycorrhizal fungi alleviate plant community instability caused by increased precipitation in arid ecosystems? Plant and Soil, 478, 559–577. 10.1007/s11104-022-05490-6

[ece310368-bib-0019] Jiang, L. , Wang, H. , Li, S. , Fu, X. , Dai, X. , Yan, H. , & Kou, L. (2021). Mycorrhizal and environmental controls over root trait‐decomposition linkage of woody trees. New Phytologist, 229(1), 284–295. 10.1111/nph.16844 32761622

[ece310368-bib-0020] Jiang, S. , Liu, Y. , Luo, J. , Qin, M. , Johnson, N. C. , Öpik, M. , Vasar, M. , Chai, Y. , Zhou, X. , Mao, L. , Du, G. , An, L. , & Feng, H. (2018). Dynamics of arbuscular mycorrhizal fungal community structure and functioning along a nitrogen enrichment gradient in an alpine meadow ecosystem. New Phytologist, 220(4), 1222–1235. 10.1111/nph.15112 29600518

[ece310368-bib-0021] Kang, F. , Yang, B. , Wu, J. , Yang, X. , Wang, L. , Guo, J. , Sun, W. , Zhang, Q. , & Zhang, T. (2020). Arbuscular mycorrhizal fungi alleviate the negative effect of nitrogen deposition on ecosystem functions in meadow grassland. Land Degradation & Development, 31(6), 748–759. 10.1002/ldr.3491

[ece310368-bib-0022] Kang, L. , Han, X. , Zhang, Z. , & Sun, O. J. (2007). Grassland ecosystems in China: Review of current knowledge and research advancement. Philosophical Transactions of the Royal Society B‐Biological Sciences, 362(1482), 997–1008. 10.1098/rstb.2007.2029 PMC2435566 17317645

[ece310368-bib-0023] Kormanik, P. P. , & Mcgraw, A. C. (1982). Quantification of vesicular‐Arbuscular mycorrhizae in plant roots. In N. C. Schenck (Ed.), Methods & principles of mycorrhizal research (pp. 37–45). American Phytopathological Society.

[ece310368-bib-0024] Lambers, H. , de Britto Costa, P. , Cawthray, G. R. , Denton, M. D. , Finnegan, P. M. , Hayes, P. E. , Oliveira, R. S. , Power, S. C. , Ranathunge, K. , Shen, Q. , Wang, X. , & Zhong, H. (2022). Strategies to acquire and use phosphorus in phosphorus‐impoverished and fire‐prone environments. Plant and Soil, 476, 133–160. 10.1007/s11104-022-05464-8

[ece310368-bib-0025] Lee, M. , Manning, P. , Rist, J. , Power, S. A. , & Marsh, C. (2010). A global comparison of grassland biomass responses to CO2 and nitrogen enrichment. Philosophical Transactions of the Royal Society B‐Biological Sciences, 365(1549), 2047–2056. 10.1098/rstb.2010.0028 PMC2880131 20513713

[ece310368-bib-0026] Lehmann, J. , & Rillig, M. (2014). Distinguishing variability from uncertainty. Nature Climate Change, 4(3), 153. 10.1038/nclimate2133

[ece310368-bib-0027] Leps, J. , Smilauerova, M. , & Smilauer, P. (2019). Competition among functional groups increases asynchrony of their temporal fluctuations in a temperate grassland. Journal of Vegetation Science, 30(6), 1068–1077. 10.1111/jvs.12803

[ece310368-bib-0028] Lin, G. , McCormack, M. L. , & Guo, D. (2015). Arbuscular mycorrhizal fungal effects on plant competition and community structure. Journal of Ecology, 103(5), 1224–1232. 10.1111/1365-2745.12429

[ece310368-bib-0029] Ma, F. , Zhang, F. , Quan, Q. , Song, B. , Wang, J. , Zhou, Q. , & Niu, S. (2021). Common species stability and species asynchrony rather than richness determine ecosystem stability under nitrogen enrichment. Ecosystems, 24(3), 686–698. 10.1007/s10021-020-00543-2

[ece310368-bib-0030] Ma, Q. , Liu, X. , Li, Y. , Li, L. , Yu, H. , Qi, M. , Zhou, G. , & Xu, Z. (2020). Nitrogen deposition magnifies the sensitivity of desert steppe plant communities to large changes in precipitation. Journal of Ecology, 108(2), 598–610. 10.1111/1365-2745.13264

[ece310368-bib-0031] Ma, Z. , Liu, H. , Mi, Z. , Zhang, Z. , Wang, Y. , Xu, W. , Jiang, L. , & He, J.‐S. (2017). Climate warming reduces the temporal stability of plant community biomass production. Nature Communications, 8(1), 15378. 10.1038/ncomms15378 PMC5436222 28488673

[ece310368-bib-0032] Ma, Z. , Zeng, Y. , Wu, J. , Zhou, Q. , & Hou, F. (2022). Plant litter influences the temporal stability of plant community biomass in an alpine meadow by altering the stability and asynchrony of plant functional groups. Functional Ecology, 36(1), 148–158. 10.1111/1365-2435.13935

[ece310368-bib-0033] Maestre, F. T. , Eldridge, D. J. , Soliveres, S. , Kéfi, S. , Delgado‐Baquerizo, M. , Bowker, M. A. , García‐Palacios, P. , Gaitán, J. , Gallardo, A. , Lázaro, R. , & Berdugo, M. (2016). Structure and functioning of dryland ecosystems in a changing world. Annual Review of Ecology, Evolution, and Systematics, 47(1), 215–237. 10.1146/annurev-ecolsys-121415-032311 PMC5321561 28239303

[ece310368-bib-0034] Maestre, F. T. , Le Bagousse‐Pinguet, Y. , Delgado‐Baquerizo, M. , Eldridge, D. J. , Saiz, H. , Berdugo, M. , Gozalo, B. , Ochoa, V. , Guirado, E. , García‐Gómez, M. , Valencia, E. , Gaitán, J. J. , Asensio, S. , Mendoza, B. J. , Plaza, C. , Díaz‐Martínez, P. , Rey, A. , Hu, H. W. , He, J. Z. , … Gross, N. (2022). Grazing and ecosystem service delivery in global drylands. Science, 378(6622), 915–920. 10.1126/science.abq4062 36423285

[ece310368-bib-0035] Ngosong, C. , Gabriel, E. , & Ruess, L. (2012). Use of the signature fatty acid 16:1ω5 as a tool to determine the distribution of arbuscular mycorrhizal fungi in soil. Journal of Lipids, 2012, 236807. 10.1155/2012/236807 22830034 PMC3398647

[ece310368-bib-0036] Olsson, P. A. , & Lekberg, Y. (2022). A critical review of the use of lipid signature molecules for the quantification of arbuscular mycorrhiza fungi. Soil Biology and Biochemistry, 166, 108574. 10.1016/j.soilbio.2022.108574

[ece310368-bib-0037] Pimm, S. L. (1984). The complexity and stability of ecosystems. Nature, 307(5949), 321–326. 10.1038/307321a0

[ece310368-bib-0038] Qiao, Y. , Bai, Y. , Zhang, Y.‐Q. , She, W. , Lai, Z. , & Qin, S. (2019). Arbuscular mycorrhizal fungi shape the adaptive strategy of plants by mediating nutrient acquisition in a shrub‐dominated community in the Mu Us Desert. Plant and Soil, 443, 549–564. 10.1007/s11104-019-04253-0

[ece310368-bib-0039] Rillig Matthias, C. , Ryo, M. , Lehmann, A. , Aguilar‐Trigueros Carlos, A. , Buchert, S. , Wulf, A. , Iwasaki, A. , Roy, J. , & Yang, G. (2019). The role of multiple global change factors in driving soil functions and microbial biodiversity. Science, 366(6467), 886–890. 10.1126/science.aay2832 31727838 PMC6941939

[ece310368-bib-0040] Sasaki, T. , Lu, X. , Hirota, M. , & Bai, Y. (2019). Species asynchrony and response diversity determine multifunctional stability of natural grasslands. Journal of Ecology, 107(4), 1862–1875. 10.1111/1365-2745.13151

[ece310368-bib-0041] Šmilauer, P. , Košnar, J. , Kotilínek, M. , Pecháčková, S. , & Šmilauerová, M. (2021). Host age and surrounding vegetation affect the community and colonization rates of arbuscular mycorrhizal fungi in a temperate grassland. New Phytologist, 232(1), 290–302. 10.1111/nph.17550 34115391

[ece310368-bib-0042] Song, J. , Wan, S. , Piao, S. , Knapp, A. K. , Classen, A. T. , Vicca, S. , Ciais, P. , Hovenden, M. J. , Leuzinger, S. , Beier, C. , Kardol, P. , Xia, J. , Liu, Q. , Ru, J. , Zhou, Z. , Luo, Y. , Guo, D. , Adam Langley, J. , Zscheischler, J. , … Zheng, M. (2019). A meta‐analysis of 1,119 manipulative experiments on terrestrial carbon‐cycling responses to global change. Nature Ecology & Evolution., 3, 1309–1320. 10.1038/s41559-019-0958-3 31427733

[ece310368-bib-0043] Song, M.‐H. , Zong, N. , Jiang, J. , Shi, P.‐L. , Zhang, X.‐Z. , Gao, J.‐Q. , Zhou, H. K. , Li, Y. K. , & Loreau, M. (2019). Nutrient‐induced shifts of dominant species reduce ecosystem stability via increases in species synchrony and population variability. Science of the Total Environment, 692, 441–449. 10.1016/j.scitotenv.2019.07.266 31351288 PMC6698194

[ece310368-bib-0044] Su, J. , Zhao, Y. , Xu, F. , & Bai, Y. (2022). Multiple global changes drive grassland productivity and stability: A meta‐analysis. Journal of Ecology, 110, 2850–2869. 10.1111/1365-2745.13983

[ece310368-bib-0045] Tilman, D. , Isbell, F. , & Cowles, J. M. (2014). Biodiversity and ecosystem functioning. Annual Review of Ecology, Evolution, and Systematics, 45(1), 471–493. 10.1146/annurev-ecolsys-120213-091917

[ece310368-bib-0046] Tilman, D. , Reich, P. B. , & Knops, J. M. H. (2006). Biodiversity and ecosystem stability in a decade‐long grassland experiment. Nature, 441(7093), 629–632. 10.1038/nature04742 16738658

[ece310368-bib-0047] Treseder, K. K. (2004). A meta‐analysis of mycorrhizal responses to nitrogen, phosphorus, and atmospheric CO_2_ in field studies. New Phytologist, 164(2), 347–355. 10.1111/j.1469-8137.2004.01159.x 33873547

[ece310368-bib-0048] Valencia, E. , de Bello, F. , Galland, T. , Adler, P. B. , Lepš, J. , E‐Vojtkó, A. , van Klink, R. , Carmona, C. P. , Danihelka, J. , Dengler, J. , Eldridge, D. J. , Estiarte, M. , García‐González, R. , Garnier, E. , Gómez‐García, D. , Harrison, S. P. , Herben, T. , Ibáñez, R. , Jentsch, A. , … Götzenberger, L. (2020). Synchrony matters more than species richness in plant community stability at a global scale. Proceedings of the National Academy of Sciences of the United States of America, 117(39), 24345–24351. 10.1073/pnas.1920405117 32900958 PMC7533703

[ece310368-bib-0049] Wagg, C. , Hautier, Y. , Pellkofer, S. , Banerjee, S. , Schmid, B. , & van der Heijden, M. G. A. (2021). Diversity and asynchrony in soil microbial communities stabilizes ecosystem functioning. eLife, 10, e62813. 10.7554/eLife.62813 33755017 PMC7987343

[ece310368-bib-0050] Wilcox, K. R. , Tredennick, A. T. , Koerner, S. E. , Grman, E. , Hallett, L. M. , Avolio, M. L. , La Pierre, K. J. , Houseman, G. R. , Isbell, F. , Johnson, D. S. , Alatalo, J. M. , Baldwin, A. H. , Bork, E. W. , Boughton, E. H. , Bowman, W. D. , Britton, A. J. , Cahill, J. F., Jr. , Collins, S. L. , Du, G. , … Zhang, Y. (2017). Asynchrony among local communities stabilises ecosystem function of metacommunities. Ecology Letters, 20(12), 1534–1545. 10.1111/ele.12861 29067791 PMC6849522

[ece310368-bib-0051] Xia, J. , & Wan, S. (2008). Global response patterns of terrestrial plant species to nitrogen addition. New Phytologist, 179(2), 428–439. 10.1111/j.1469-8137.2008.02488.x 19086179

[ece310368-bib-0052] Xu, Q. , Yang, X. , Yan, Y. , Wang, S. , Loreau, M. , & Jiang, L. (2021). Consistently positive effect of species diversity on ecosystem, but not population, temporal stability. Ecology Letters, 24(10), 2256–2266. 10.1111/ele.13777 34002439

[ece310368-bib-0053] Xu, Z. , Ren, H. , Li, M.‐H. , van Ruijven, J. , Han, X. , Wan, S. , Li, H. , Yu, Q. , Jiang, Y. , & Jiang, L. (2015). Environmental changes drive the temporal stability of semi‐arid natural grasslands through altering species asynchrony. Journal of Ecology, 103(5), 1308–1316. 10.1111/1365-2745.12441

[ece310368-bib-0054] Yang, G. (2021). Plant and soil biodiversity have non‐substitutable stabilizing effects on biomass production. Ecology Letters, 24, 1582–1593. 34053155 10.1111/ele.13769

[ece310368-bib-0055] Yang, G. , Liu, N. , Lu, W. , Wang, S. , Kan, H. , Zhang, Y. , Xu, L. , & Chen, Y. (2014). The interaction between arbuscular mycorrhizal fungi and soil phosphorus availability influences plant community productivity and ecosystem stability. Journal of Ecology, 102(4), 1072–1082. 10.1111/1365-2745.12249

[ece310368-bib-0056] Yang, G. , Wagg, C. , Veresoglou, S. D. , Hempel, S. , & Rillig, M. C. (2018). How soil biota drive ecosystem stability. Trends in Plant Science, 23(12), 1057–1067. 10.1016/j.tplants.2018.09.007 30287162

[ece310368-bib-0057] Yang, G. , Zhang, Y. , Yang, X. , Liu, N. , Rillig, M. C. , Veresoglou, S. D. , & Wagg, C. (2021). Mycorrhizal suppression and phosphorus addition influence the stability of plant community composition and function in a temperate steppe. Oikos, 130(3), 354–365. 10.1111/oik.07610

[ece310368-bib-0058] Yang, G.‐J. , Hautier, Y. , Zhang, Z.‐J. , Lü, X.‐T. , & Han, X.‐G. (2022). Decoupled responses of above‐ and below‐ground stability of productivity to nitrogen addition at the local and larger spatial scale. Global Change Biology, 28, 2711–2720. 10.1111/gcb.16090 35098614

[ece310368-bib-0059] Yang, H. , Jiang, L. , Li, L. , Li, A. , Wu, M. , & Wan, S. (2012). Diversity‐dependent stability under mowing and nutrient addition: Evidence from a 7‐year grassland experiment. Ecology Letters, 15(6), 619–626. 10.1111/j.1461-0248.2012.01778.x 22487498

[ece310368-bib-0060] Yang, X. , Liu, Y. , Tian, H. , & Shen, Y. (2021). Short‐term nitrogen and phosphorus additions rather than mycorrhizal suppression determine plant community composition and productivity in desert steppe. Applied Soil Ecology, 168, 104144. 10.1016/j.apsoil.2021.104144

[ece310368-bib-0061] Yang, X. , Mariotte, P. , Guo, J. , Hautier, Y. , & Zhang, T. (2021). Suppression of arbuscular mycorrhizal fungi decreases the temporal stability of community productivity under elevated temperature and nitrogen addition in a temperate meadow. Science of the Total Environment, 762, 143137. 10.1016/j.scitotenv.2020.143137 33121784

[ece310368-bib-0062] Yang, X. , Shen, Y. , Liu, N. , Wilson, G. W. T. , Cobb, A. B. , & Zhang, Y. (2018). Defoliation and arbuscular mycorrhizal fungi shape plant communities in overgrazed semiarid grasslands. Ecology, 99(8), 1847–1856. 10.1002/ecy.2401 29845596

[ece310368-bib-0063] Zhang, Y. , He, N. , Loreau, M. , Pan, Q. , & Han, X. (2018). Scale dependence of the diversity–stability relationship in a temperate grassland. Journal of Ecology, 106(3), 1277–1285. 10.1111/1365-2745.12903 PMC5916871 29725139

[ece310368-bib-0064] Zhang, Y. , Loreau, M. , Lü, X. , He, N. , Zhang, G. , & Han, X. (2016). Nitrogen enrichment weakens ecosystem stability through decreased species asynchrony and population stability in a temperate grassland. Global Change Biology, 22(4), 1445–1455. 10.1111/gcb.13140 26511538

[ece310368-bib-0065] Zhou, B. , Li, S. , Li, F. , Dong, S. , Ma, F. , Zhu, S. , Zhou, H. , & Stufkens, P. (2019). Plant functional groups asynchrony keep the community biomass stability along with the climate change‐ a 20‐year experimental observation of alpine meadow in eastern Qinghai‐Tibet plateau. Agriculture, Ecosystems & Environment, 282, 49–57. 10.1016/j.agee.2019.06.002

[ece310368-bib-0066] Zhou, G. , Luo, Q. , Chen, Y. , He, M. , Zhou, L. , Frank, D. , He, Y. , Fu, Y. , Zhang, B. , & Zhou, X. (2019). Effects of livestock grazing on grassland carbon storage and release override impacts associated with global climate change. Global Change Biology, 25(3), 1119–1132. 10.1111/gcb.14533 30466147

[ece310368-bib-0067] Zhou, M. , Yang, Q. , Zhang, H. , Yao, X. , Zeng, W. , & Wang, W. (2020). Plant community temporal stability in response to nitrogen addition among different degraded grasslands. Science of the Total Environment, 729, 138886. 10.1016/j.scitotenv.2020.138886 32361447

