# Supplementary material for: Negative effects of phosphorus addition outweigh effects of arbuscular mycorrhizal fungi and nitrogen addition on grassland temporal stability in the eastern Eurasian desert steppe
Source: Ecol Evol. 2023 Aug 4;13(8):e10368. doi: 10.1002/ece3.10368 (PMC10401164; doi:10.1002/ece3.10368)
Supplement: Supplementary file 1 — Appendix S1–S8 [file ECE3-13-e10368-s001.docx]

**Negative effects of phosphorus addition outweigh effects of arbuscular mycorrhizal fungi and nitrogen addition on grassland temporal stability in eastern Eurasian desert steppe**

**Appendix**

**Appendix S1:** Monthly distribution of precipitation from 2019 to 2021.

**Appendix S2:** Plant species found in the study sites and their type and proportion.

**Appendix S3:** Analysis of variance for the effects of year (Y), fungicide application (F), nitrogen addition (N) and phosphorus addition (P) on the shoot biomass of plant species from 2019 to 2021.

**Appendix S4:** Analysis of variance for the effects of year (Y), fungicide application (F), nitrogen addition (N) and phosphorus addition (P) on plant species richness and Shannon diversity index from 2019 to 2021.

**Appendix S5:** Analysis of variance for the effects of year (Y), fungicide application (F), nitrogen addition (N) and phosphorus addition (P) on neutral fatty acid concentrations in 2019 and 2021.

**Appendix S6:** Response of soil available N and soil available P to nitrogen addition (N) and phosphorus addition (P) in no fungicide addition (dark green bars) and fungicide addition (light green bars) plots from 2019 to 2021.

**Appendix S7:** Response of mycorrhizal root colonisation (%) to nitrogen addition (N) and phosphorus addition (P) in no fungicide addition (dark green bars) and fungicide addition (light green bars) plots from 2019 to 2021.

**Appendix S8:** Response of AM fungal NLFA concentration (nmol g^−1^ soil) to nitrogen addition (N) and phosphorus addition (P) in no fungicide addition (dark green bars) and fungicide addition (light green bars) plots from 2019 to 2021.


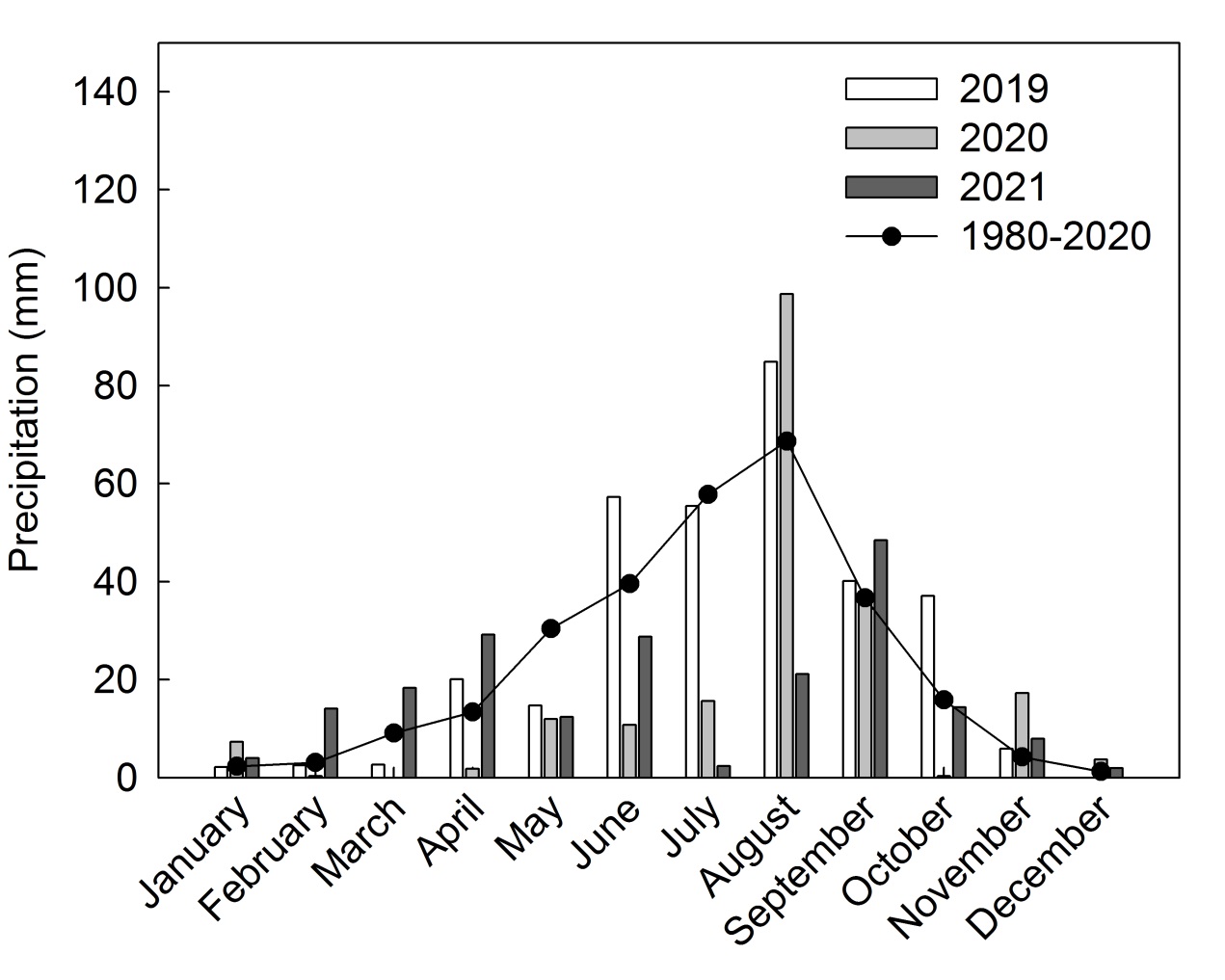


**Appendix S1.** Monthly distribution of precipitation from 2019 to 2021.

**Appendix S2.** Plant species found in the study sites and their type and proportion. Proportion (%) = average aboveground biomass of plant species / total aboveground biomass.

| Plant species | Plant functional group | Life form | Proportion (%) |
| --- | --- | --- | --- |
| *_Stipa breviflora_* | C_3_ grass | Perennial | 28.91 |
| *_Agropyron mongolicum_* | C_3_ grass | Perennial | 13.66 |
| *_Cleistogenes squarrosa_* | C_4_ grass | Perennial | 7.87 |
| *_Chenopodium aristatum_* | Forb | Annual | 9.54 |
| *_Salsola collina_* | Forb | Annual | 1.66 |
| *_Cynanchum komarovii_* | Forb | Perennial | 4.43 |
| *_Allium mongolicum_* | Forb | Perennial | 0.53 |
| *_Allium tenuissimum_* | Forb | Perennial | 1.25 |
| *_Polygala tenuifolia_* | Forb | Perennial | 3.94 |
| *_Torularia humilis_* | Forb | Perennial | 2.57 |
| *_Convolvulus ammannii_* | Forb | Perennial | 9.16 |
| *_Euphorbia esula_* | Forb | Perennial | 0.37 |
| *_Ixeridium gracile_* | Forb | Perennial | 0.44 |
| *_Heteropappus altaicus_* | Forb | Perennial | 0.06 |
| *_Astragalus melilotoides_* | Legume | Perennial | 9.68 |
| *_Lespedeza potaninii_* | Legume | Perennial | 5.61 |
| *_Oxytropis racemosa_* | Legume | Perennial | 0.28 |

*Notes*: Legumes are the only kinds of N_2_-fixing forbs.

**Appendix S3.** Analysis of variance for the effects of year (Y), fungicide application (F), nitrogen addition (N) and phosphorus addition (P) on the shoot biomass of plant species from 2019 to 2021.

|  |  | Community biomass | |  | C_3_ grasses | |  | C_4_ grasses | |  | Non-N_2_-fixing forbs | |  | N_2_-fixing forbs | |
| --- | --- | --- | --- | --- | --- | --- | --- | --- | --- | --- | --- | --- | --- | --- | --- |
| *Effects* | *d.f.* | *F* | *P* |  | *F* | *P* |  | *F* | *P* |  | *F* | *P* |  | *F* | *P* |
| Between-subject | |  |  |  |  |  |  |  |  |  |  |  |  |  |  |
| Block | 4, 28 | 0.51 | 0.73 |  | 0.37 | 0.83 |  | 1.07 | 0.50 |  | 1.67 | 0.18 |  | 0.30 | 0.88 |
| F | 1, 28 | **15.67** | **< 0.01** |  | **16.43** | **< 0.01** |  | 0.40 | 0.57 |  | **5.04** | **0.03** |  | 1.64 | 0.21 |
| N | 1, 28 | **20.58** | **< 0.01** |  | **8.49** | **0.01** |  | **13.08** | **0.04** |  | **22.79** | **< 0.01** |  | 0.22 | 0.64 |
| P | 1, 28 | **157.60** | **< 0.01** |  | **122.48** | **< 0.01** |  | 8.49 | 0.06 |  | **7.29** | **0.01** |  | **28.47** | **< 0.01** |
| F × N | 1, 28 | 1.22 | 0.28 |  | 1.96 | 0.17 |  | 0.09 | 0.78 |  | 0.41 | 0.53 |  | 2.42 | 0.13 |
| F ×P | 1, 28 | **15.40** | **< 0.01** |  | **17.48** | **< 0.01** |  | 0.02 | 0.90 |  | 1.30 | 0.26 |  | 0.09 | 0.77 |
| N × P | 1, 28 | **5.12** | **0.03** |  | **4.02** | **0.05** |  | 4.48 | 0.12 |  | 3.22 | 0.08 |  | 1.13 | 0.30 |
| F × N × P | 1, 28 | 1.02 | 0.32 |  | **6.39** | **0.02** |  | 1.07 | 0.50 |  | **4.93** | **0.03** |  | 1.29 | 0.26 |
| Within-subject | | |  |  |  |  |  |  |  |  |  |  |  |  |  |
| Y | 1, 28 | **41.12** | **< 0.01** |  | **149.23** | **< 0.01** |  | **106.60** | **< 0.01** |  | **80.89** | **< 0.01** |  | 0.20 | 0.66 |
| Y×F | 1, 28 | **18.46** | **< 0.01** |  | **24.90** | **< 0.01** |  | 3.49 | 0.16 |  | 0.10 | 0.76 |  | 2.39 | 0.13 |
| Y×N | 1, 28 | 0.07 | 0.80 |  | **4.59** | **0.04** |  | **39.56** | **0.01** |  | **7.99** | **0.01** |  | **8.00** | **0.01** |
| Y×P | 1, 28 | **20.74** | **< 0.01** |  | **90.01** | **< 0.01** |  | **14.77** | **0.03** |  | **68.55** | **< 0.01** |  | 0.19 | 0.67 |
| Y×F×N | 1, 28 | 1.08 | 0.31 |  | 0.80 | 0.38 |  | 0.15 | 0.72 |  | 0.00 | 0.98 |  | 1.35 | 0.26 |
| Y×F × P | 1, 28 | **13.69** | **< 0.01** |  | **20.31** | **< 0.01** |  | 0.62 | 0.49 |  | 0.90 | 0.35 |  | 0.11 | 0.74 |
| Y×N × P | 1, 28 | 1.46 | 0.24 |  | 1.79 | 0.19 |  | **14.91** | **0.03** |  | **6.00** | **0.02** |  | **19.53** | **< 0.01** |
| Y×F × N × P | 1, 28 | 0.86 | 0.36 |  | 1.49 | 0.23 |  | 3.49 | 0.16 |  | 0.02 | 0.90 |  | 0.01 | 0.93 |

*Notes*: ANPP and the aboveground biomass of plant functional group all have the same d.f. Bold values are significant at *P* < 0.05.

**Appendix S4.** Analysis of variance for the effects of year (Y), fungicide application (F), nitrogen addition (N) and phosphorus addition (P) on plant species richness and Shannon diversity index from 2019 to 2021.

|  |  | Plant species richness | |  | Shannon diversity index | |
| --- | --- | --- | --- | --- | --- | --- |
| *Effects* | *d.f.* | *F* | *P* |  | *F* | *P* |
| Between-subject | |  |  |  |  |  |
| Block | 4, 28 | 0.86 | 0.50 |  | 0.67 | 0.62 |
| F | 1, 28 | 0.21 | 0.65 |  | 1.86 | 0.18 |
| N | 1, 28 | 0.42 | 0.52 |  | 1.53 | 0.23 |
| P | 1, 28 | **13.04** | **< 0.01** |  | **9.03** | **0.01** |
| F × N | 1, 28 | 2.19 | 0.15 |  | 0.20 | 0.65 |
| F ×P | 1, 28 | **4.94** | **0.03** |  | 2.12 | 0.16 |
| N × P | 1, 28 | 1.23 | 0.28 |  | 0.41 | 0.53 |
| F × N × P | 1, 28 | 1.93 | 0.18 |  | 3.08 | 0.09 |
| Within-subject | | |  |  |  |  |
| Y | 1, 28 | **72.38** | **< 0.01** |  | **164.04** | **< 0.01** |
| Y×F | 1, 28 | 0.28 | 0.60 |  | 1.60 | 0.22 |
| Y×N | 1, 28 | 0.44 | 0.51 |  | 3.87 | 0.06 |
| Y×P | 1, 28 | **12.88** | **< 0.01** |  | **61.30** | **< 0.01** |
| Y×F×N | 1, 28 | 0.44 | 0.51 |  | 0.00 | 0.98 |
| Y×F × P | 1, 28 | 0.16 | 0.69 |  | 0.15 | 0.70 |
| Y×N × P | 1, 28 | 1.13 | 0.30 |  | **4.88** | **0.04** |
| Y×F × N × P | 1, 28 | 1.77 | 0.19 |  | 4.56 | 0.06 |

*Notes*: Treatments were fungicide (F), N and P addition. Plant species richness and Shannon diversity index all have the same d.f. Bold values are significant at *P* < 0.05.

**Appendix S5.** Analysis of variance for the effects of year (Y), fungicide application (F), nitrogen addition (N) and phosphorus addition (P) on neutral fatty acid concentrations in 2019 and 2021.

|  |  | AM Fungi | |  | Saprophytic Fungi | |  | Non-specific Microbes | |  | Gram^+^ - Bacteria | |  | Gram^-^ - Bacteria | |
| --- | --- | --- | --- | --- | --- | --- | --- | --- | --- | --- | --- | --- | --- | --- | --- |
| *Effects* | *d.f.* | *F* | *P* |  | *F* | *P* |  | *F* | *P* |  | *F* | *P* |  | *F* | *P* |
| Between-subject | |  |  |  |  |  |  |  |  |  |  |  |  |  |  |
| Block | 4, 28 | 0.29 | 0.88 |  | 9.81 | 0.00 |  | 1.29 | 0.30 |  | 1.78 | 0.16 |  | 1.88 | 0.14 |
| F | 1, 28 | **87.17** | **< 0.01** |  | 1.15 | 0.29 |  | 0.00 | 0.96 |  | 0.04 | 0.85 |  | 1.85 | 0.18 |
| N | 1, 28 | 0.01 | 0.94 |  | 0.34 | 0.56 |  | 1.21 | 0.28 |  | 2.34 | 0.14 |  | 1.84 | 0.21 |
| P | 1, 28 | **24.96** | **< 0.01** |  | 1.13 | 0.30 |  | 0.40 | 0.53 |  | 0.05 | 0.83 |  | 1.41 | 0.24 |
| F × N | 1, 28 | 0.02 | 0.89 |  | 0.07 | 0.80 |  | 0.90 | 0.35 |  | 0.01 | 0.92 |  | 0.00 | 0.99 |
| F ×P | 1, 28 | 0.43 | 0.52 |  | 0.46 | 0.51 |  | 1.16 | 0.29 |  | 0.03 | 0.86 |  | 0.00 | 0.96 |
| N × P | 1, 28 | **5.68** | **0.02** |  | 0.07 | 0.79 |  | 2.05 | 0.16 |  | 1.01 | 0.32 |  | 0.05 | 0.83 |
| F × N × P | 1, 28 | 0.36 | 0.56 |  | 0.01 | 0.92 |  | 0.23 | 0.63 |  | 0.01 | 0.90 |  | 1.45 | 0.24 |
| Within-subject | | |  |  |  |  |  |  |  |  |  |  |  |  |  |
| Y | 1, 28 | 1.00 | 0.63 |  | 8.31 | 0.01 |  | **42.58** | **< 0.01** |  | **80.09** | **< 0.01** |  | **10.53** | **< 0.01** |
| Y×F | 1, 28 | 0.84 | 0.37 |  | 1.38 | 0.25 |  | 1.11 | 0.30 |  | 0.07 | 0.79 |  | **14.02** | **< 0.01** |
| Y×N | 1, 28 | 0.02 | 0.89 |  | 0.38 | 0.54 |  | **5.70** | **0.02** |  | 2.40 | 0.13 |  | 0.45 | 0.51 |
| Y×P | 1, 28 | 2.28 | 0.14 |  | 0.25 | 0.62 |  | 0.46 | 0.50 |  | 0.02 | 0.88 |  | **5.65** | **0.02** |
| Y×F×N | 1, 28 | 1.31 | 0.26 |  | 0.68 | 0.42 |  | 1.48 | 0.23 |  | 0.20 | 0.66 |  | 0.16 | 0.70 |
| Y×F × P | 1, 28 | 0.66 | 0.42 |  | 1.45 | 0.24 |  | 0.68 | 0.42 |  | 0.07 | 0.79 |  | 0.40 | 0.53 |
| Y×N × P | 1, 28 | 0.09 | 0.77 |  | 2.76 | 0.11 |  | **9.35** | **< 0.01** |  | 1.11 | 0.30 |  | **9.64** | **< 0.01** |
| Y×F × N × P | 1, 28 | 0.35 | 0.56 |  | **< 0.01** | 0.98 |  | 1.12 | 0.30 |  | 0.05 | 0.82 |  | 1.94 | 0.17 |

*Notes*: Treatments were fungicide (F), N and P addition. AM Fungi, Saprophtic fungi, Non-specific microbes, Gram^+^- bacteria, Gram^–^- bacteria all have the same *d.f*. Bold values are significant at *P* < 0.05.


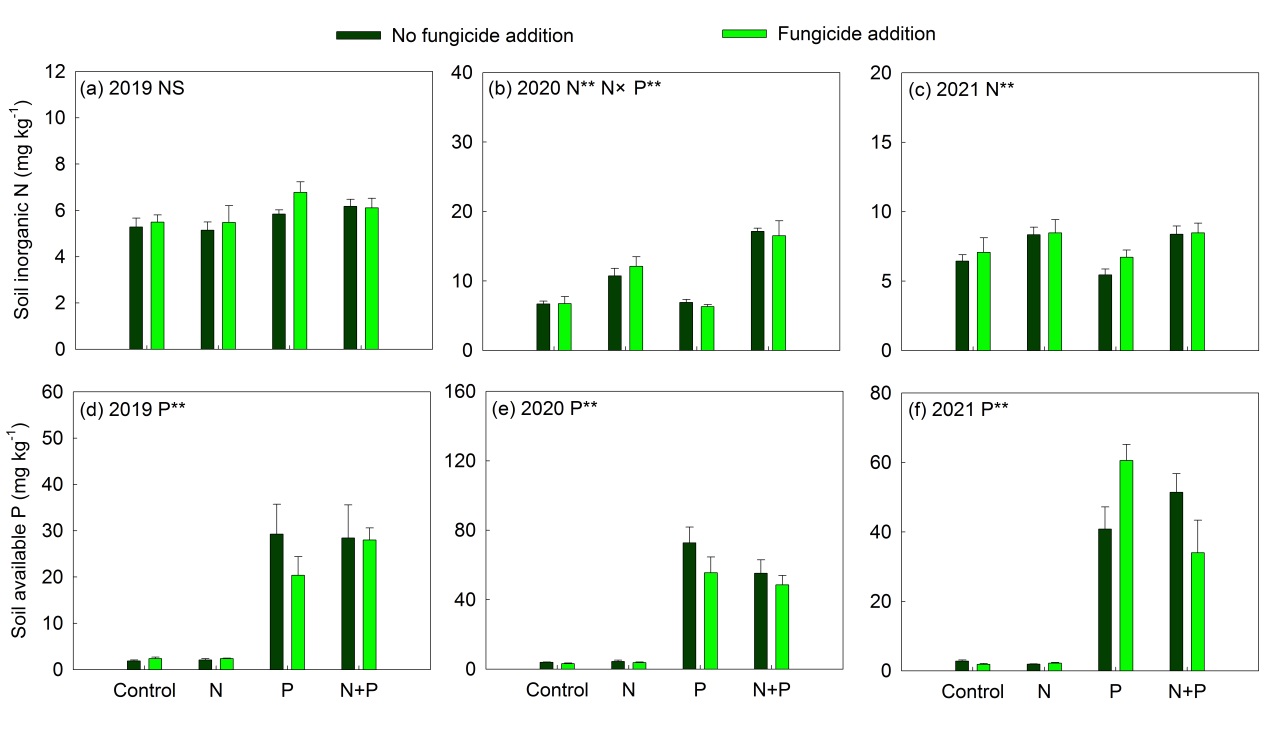


**Appendix S6**. Response of soil available N (a, b) and soil available P (c, d) to nitrogen addition (N) and phosphorus addition (P) in no fungicide addition (dark green bars) and fungicide addition (light green bars) plots from 2019 to 2021. Data presented as means + SE. ***p* < 0.01; NS *p* > 0.05.


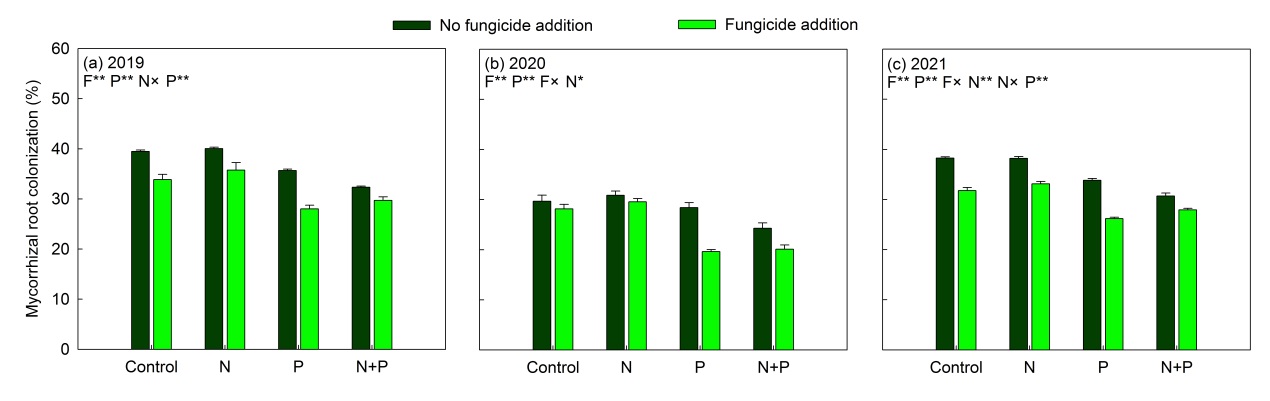


**Appendix S7.** Response of mycorrhizal root colonisation (%) to nitrogen addition (N) and phosphorus addition (P) in no fungicide addition (dark green bars) and fungicide addition (light green bars) plots from 2019 to 2021. F, fungicide application. Data presented as means + SE. ***p* < 0.01; NS *p* > 0.05.


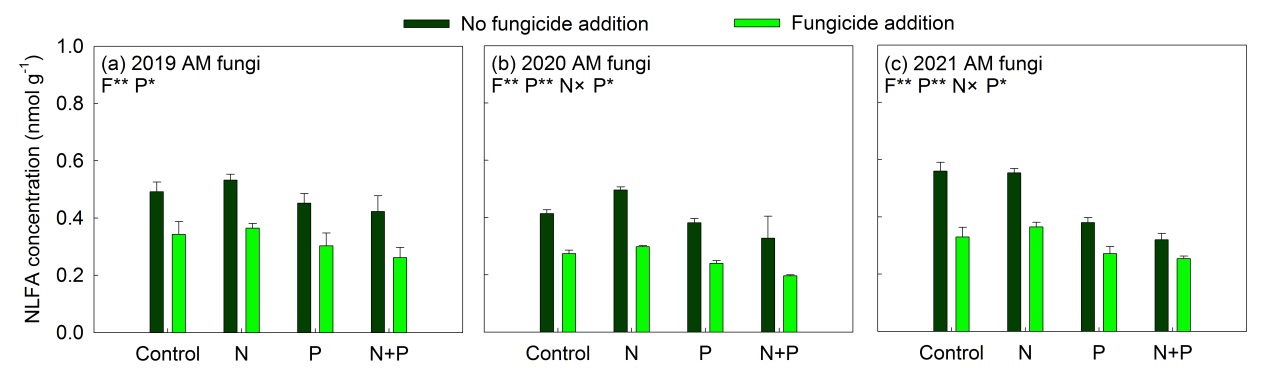


**Appendix S8.** Response of AM fungal NLFA concentration (nmol g^−1^ soil) to nitrogen addition (N) and phosphorus addition (P) in no fungicide addition (dark green bars) and fungicide addition (light green bars) plots from 2019 to 2021. F, fungicide application. Data presented as means + SE. ***p* < 0.01; NS *p* > 0.05.
